# Metabolic rate and substrate utilisation resilience in men undertaking polar expeditionary travel

**DOI:** 10.1371/journal.pone.0221176

**Published:** 2019-08-15

**Authors:** John Hattersley, Adrian J. Wilson, C. Doug Thake, Jamie Facer-Childs, Oliver Stoten, Chris Imray

**Affiliations:** 1 Coventry NIHR CRF Human Metabolic Research Unit, University Hospitals Coventry and Warwickshire NHS Trust, Coventry, United Kingdom; 2 School of Engineering, University of Warwick, Coventry, United Kingdom; 3 Faculty of Health and Life Sciences, Coventry University, Coventry, United Kingdom; 4 Department of Physics, University of Warwick, Coventry, United Kingdom; 5 Institute of Child Health, University College London, London, United Kingdom; 6 Emergency Department, Royal Bournemouth Hospital, Bournemouth, Uinted Kingdom; 7 Department of Vascular and Renal Transplant Surgery, University Hospitals Coventry and Warwickshire NHS Trust, Coventry, United Kingdom; California State University San Marcos, UNITED STATES

## Abstract

The energy expenditure and substrate utilisation were measured in 5 men pre- and post- a 67 day, 1750km unassisted Antarctic traverse from the Hercules Inlet to the Ross Sea Ice via the South pole pulling sledges weighing 120kg whilst experiencing temperatures as low as -57°C. A 36-hours protocol in a whole body calorimeter was employed to measure periods of rest, sleep and three periods of standardised stepping exercises at 80, 100 and 120 steps min^-1^; participants were fed isocalorically. Unlike previous expeditions where large weight loss was reported, only a modest loss of body weight (7%, *P =* 0.03) was found; fat tissue was reduced by 53% (*P =* 0.03) together with a small, but not statistically significant, increase in lean tissue weight (*P* = 0.18). This loss occurred despite a high-energy intake (6500 kcal/day) designed to match energy expenditure. An energy balance analysis suggested the loss in body weight could be due to the energy requirements of thermoregulation. Differences in energy expenditure [4.9 (0.1) vs 4.5 (0.1) kcal/min. *P* = 0.03], carbohydrate utilisation [450 (180) vs 569 (195) g/day; *P* = 0.03] and lipid utilisation [450 (61) vs 388 (127) g/day, *P* = 0.03] at low levels of exertion were different from pre-expedition values. Only carbohydrate utilisation remained statistically significant when normalised to body weight. The differences in energy expenditure and substrate utilisation between the pre- and post-expedition for other physiological states (sleeping, resting, higher levels of exercise, etc) were small and not statistically significant. Whilst inter-subject variability was large, there was a tendency for increased carbohydrate utilisation, post-expedition, when fasted that decreased upon feeding.

## Introduction

Body weight loss is common in expeditionary travel as a result of negative energy availability (EA) where energy expenditure required for travel exceeds the nutritional energy intake which is evidenced by a loss of body weight [1–10]. Small levels of weight loss are seen in those undertaking low levels of work in extreme environments [11], whereas it is participants in unsupported polar expeditions that have high levels of negative energy availability [2–4, 12, 13], with up to 25% loss of body weight being reported in the literature [1].

Capturing information on energy expenditure during polar exploration is challenging and the number of participants limited by the size of the expeditionary team. Whilst indirect calorimetry has been used to measure the metabolic rate in polar environments [14] and in the laboratory where aspects of the polar environment have been simulated [15], it has been argued that the Doubly Labelled Water (DLW) technique [16], is the only realistic method of making energy expenditure measurements during the expedition itself [17]. Both DLW and another isotope dilution technique using ^15^N to measure protein metabolism [18] have been used during polar expeditions to measure the energy expenditure [1, 3, 4]. Isotope methods, by their nature, give a time-averaged measurement of the metabolic rate. However, a standardized daily routine during an expedition is the norm and therefore isotope methods have the potential to give a robust estimate of the metabolic rate. It should be noted that using DLW for studies where the participants move geographic location presents a challenge for the technique, as local water for drinking and cooking will have different abundance of the isotopes used for measurement [3, 4, 19] resulting in problems in accurately determining the baseline levels of the isotopes. Polar ice has been reported as having lower concentrations of the hydrogen and oxygen isotopes used in DLW measurements than standard water supplies [3]. The procedures proposed by Stroud *et al*. (3) have been successfully used to establish baseline levels during expeditionary polar journeys [3, 4]. Previous studies of metabolic energy expenditure during polar expeditions using isotope dilution techniques have reported a measured energy expenditure substantially higher than that predicted by activity [15] and a 60% increase in basal metabolic rate attributed to thyroid activity [2]. This increase in basal metabolic rate due to the cold and altitude is supported by the reduction in body weight seen by those over-wintering in Antarctica who are only undertaking light work and have a diet appropriate to the level of activity [11].

What isotope methods cannot do is measure the change in the metabolic rate and substrate utilisation because of the expedition. In this paper we use indirect calorimetry measured in a respiratory chamber [20] pre- and post- an expeditionary journey in Antarctica to investigate the change in diurnal energy expenditure and substrate oxidation.

During the Antarctic summer of 2016/17 a team of six British Army Reservists, including an experienced polar traveller who was their leader, undertook an unassisted crossing of Antarctica (https://www.forces.net/news/feature/spear-17-team-complete-mammoth-antarctic-expedition). The 67 day, 1750km journey took them from the Hercules Inlet to the South Pole and then down the Shackleton Glacier, finally finishing on the Ross Sea Ice. During the journey, they experienced temperatures as low as -57 C whilst pulling sledges with a planned maximum weight of 120kg to an altitude of 3350m. The daily food intake and macronutrient composition during the expedition was designed pre-expedition to be 6,500 kcal (27.2 MJ), with a macronutrient breakdown of 55% fat, 37% carbohydrate and 8% protein; this calculation was based on an increase from previous expeditions of a similar nature in which weight loss was seen [Frykman *et al*. (10) *-* 25.1 MJ.day-1, (6000 kCals.day-1); Stroud *et al*. (4)*-* 5091 kCals.day-1 (21.3 MJ.day-1)]. Subjects consumed a freeze-dried meal at breakfast, shortly followed by 1L hot chocolate. A mixture of chocolate, nuts, energy bars and dried fruit was consumed ad arbitrium during the day; a day which consisted of cycles of 70 minutes sledge hauling followed by a 10 minute break. At the end of the day subjects consumed a meal-replacement drink (CNP Professional, Hyde, UK) containing 484 kCals (21g fat, 20.6g protein, 52.5g carbohydrate). A freeze-dried dinner and pudding (Expedition Foods, Hull, UK) followed this later in the evening. During the post-expedition studies in the whole-body calorimeters, the percentage of the food consumed during the expedition was estimated by each participant, from this information the average daily food consumption was estimated to be between 92% (70%-99%) of what was provided. Anecdotally, the participants indicated that there was minimal food swapping for palatability during the expedition. It must be stated that the development of the diet was not part of the study, this was conducted exclusively by the expeditionary team but it is reported here for completeness.

By their very nature, the number of subjects undertaking expeditionary journeys in hostile environments is limited but there are an increasing number of polar expeditions, both civilian and military, being undertaken. To maximise the benefit of research in this area a central repository is being created, the Global Polar Altitude Metabolic Research Registry (https://www.rgs.org/in-the-field/advice-training/resources-for-expeditions/global-polar-altitude-metabolic-research-register), to collect data on subjects undertaking such expeditions. This is an ongoing longitudinal registry study designed to gain a better insight into the physiological and metabolic changes in individuals undertaking extended expeditions to polar and high altitude environments. The longer term aim is to use these metabolic challenges in cold and/or hypoxic environments as a potential surrogate for the catabolic challenges associated with diseases and their treatments [21].

## Subject and methods

### Study population

The six male participants in the expedition (median (IQR) age 29 (8)) were invited to participate in the scientific study of the impact of the expedition on their metabolism. Participation in the scientific study was voluntary and independent of their participation in the expedition. All six men volunteered to be participants in the scientific study and gave written consent prior to any data collection; unfortunately, one member of the team was unable to complete the expedition and only attended the pre-expedition measurement session. The data from this individual has been excluded from the study. Ethical approval for this study was obtained from National Health Authority Research Ethics Committee, West Midlands—Solihull (ID: 13/WM/0327), Ministry of Defence—Research Ethics Committee and University Hospitals Coventry and Warwick Research and Development Governance Committee, under the GAFREC framework (REF: GF0121). This study was compliant with the Ethical Principles for Medical Research on Human Subjects set down in the Declaration of Helsinki by the World Medical Association.

### Calorimetry

The participants underwent metabolic studies twice: the first in the two weeks prior to departure from the UK and the second within two weeks following their return to the UK; it must be noted that this does not equate to two weeks pre- and post-expedition, the team spent 2 weeks in transit to and from Antarctica. The central component of each investigation was a 36-hour measurement in a whole body calorimeter starting 21:30. The first evening and night were regarded as the ‘acclimatisation period’ when the participants got used to the environment and the following 24 hours starting at 07:00 designated as the ‘measurement period’. The experimental facility has two whole body calorimeters each of which is a gas-tight and thermally insulated small self-contained living space (floor dimensions 2.9m x 2.1m) containing a bed, desk, chair and freezer toilet [20, 22]. From the difference between the concentration of O_2_ and CO_2_ levels entering and leaving the calorimeter the metabolic rate, carbohydrate and lipid utilisation were determined using the Brouwer formulae [23, 24]. Protein metabolism was determined from the nitrogen in urine samples taken over 12 hour periods [25]. Each chamber is fitted with two double door ports, one for food and liquids, the other for waste, so that the environment and gas concentration of the chamber were minimally affected during the necessary passage of items into and out of the chambers. An ultrasound detector within the room (bespoke product, Maastricht Instruments, Nl) recorded the participant’s movement. Windows, with blinds operated from within the chamber, allowed a participant to see out into the laboratory and the participant in the other chamber. Vocal communication with both the other participant and the researchers was through an intercom. To maintain thermal neutrality, the environment within the chamber was controlled at a relative humidity of 57±5% at a temperature of 24±0.5°C during the day and 22±0.5°C during the night.

**Fig 1:**
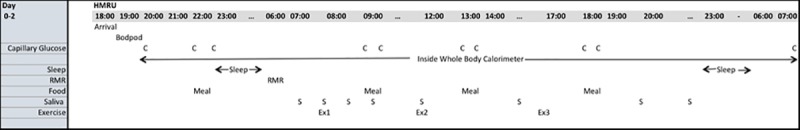
The protocol for the measurements in the metabolic chamber. It should be noted that the capillary blood sampling (C) and the saliva sampling (S) were both carried out by the participants.

During the period in the calorimeters the food intake of the participants was isocaloric, calculated from lean tissue mass. The composition of the food was based on a typical western diet (50% carbohydrate, 35% fat and 15% protein). The diet contained no tea, coffee, caffeinated beverages or alcohol but water and non-caffeinated herbal teas were available *ad libitum*. The study protocol is shown diagrammatically in Fig 1. For completeness, saliva sampling and blood glucose determination from capillary samples are shown, but only the results for the energetic measurements are reported in this paper. Three 30-minute periods of stepping exercise using a standard height exercise step (Reebok Aerobic Step, height 150mm, Reebok, UK) were undertaken at step rates of 80, 100 and 120 steps min^-1^, with the step rate paced by a simple metronome (Tempo Perfect, https://www.nch.com.au/metronome).

### Body composition

Prior to entering the calorimeters, the participants had their height and weight measured (Seca 799, Seca UK) and then their body composition determined by Air Displacement Plethysmography (ADP) [26] using a BodPod 2000A (Cosmed Inc., USA). The scales linked to the BODPod were calibrated weekly using 20 kg standards; the BODPod was calibrated daily using a 50.034 L test volume. In line with the manufacturers’ protocol, subjects wore minimal tight fitting garments (e.g. swimming costume and cap) and voided their bladder before measurement to minimise isothermic and water retention errors.

### Urinary analysis

Urine was collected over three 12-hour periods with participants encouraged to void before entering the room. Collected urine volume was measured using digital weighing scales (Salter 323, Salter, UK) and analysed enzymatically for urea and creatinine (UREA and CREAT kits, respectively from Roche, DE) using an automated clinical chemistry analyser (Cobas c702, Roche, DE).

### Data analysis

**Fig 2:**
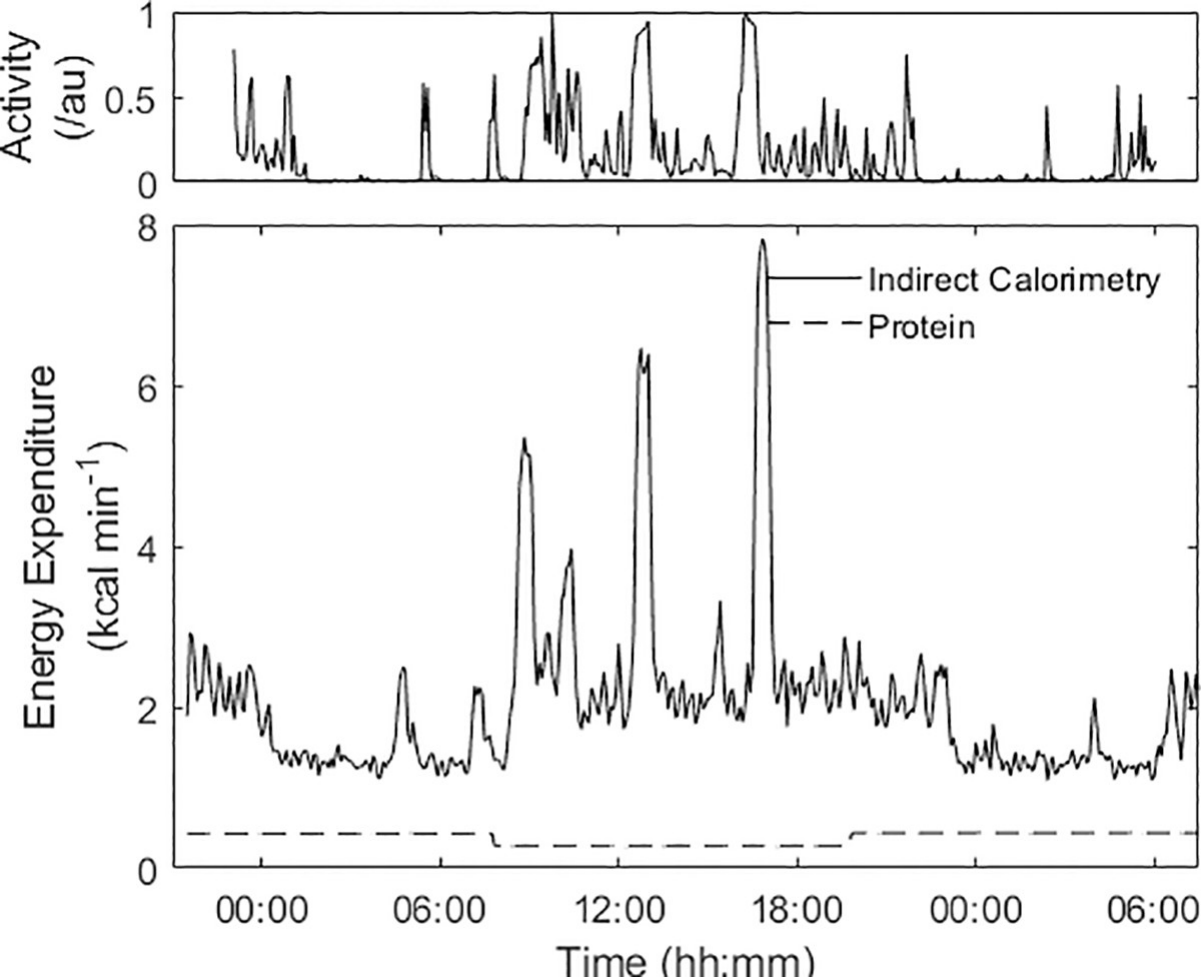
An example of the time history of energy expenditure during the 36-hour study period. The glucose/glycogen energy expenditure is determined from the difference in the O2 and CO2 concentrations entering and leaving the whole body calorimeter. The protein energy is determined from the 12-hour urine sample analysis. The participant activity from the ultrasound movement sensors has been scaled 0–1 and is shown at the top of the graph.

The metabolic rate is determined on a minute-by-minute basis from the continuous measurements of the difference in concentration of O_2_ and CO_2_ in the gases entering and leaving the room. This, together with the data from the movement sensors, gave a profile of the energy expenditure and activity for the participant over the study period as shown in Fig 2. The figure also shows the energy due to protein metabolism where expenditure was estimated from the analysed creatinine and urea in the 12-hour urine samples [25]. The total energy expenditure is the sum of the energy expenditure from the O_2_ and CO_2_ measurements, and the protein from the urine nitrogen analysis. From these data the following factors were determined:

### Total energy expenditure (TEE)

The sum of all energy expenditure for the 24-hour measurement period.

### Sleeping metabolic rate (SMR)

Two values were determined, one from each of the two periods of sleep. The metabolic rate during sleep is determined by the amount of metabolically active tissue in the body so both raw values and the values normalised to the weight of lean tissue were compared between participants.

### Resting metabolic rate (RMR)

The resting metabolic rate was measured between 07:00 and 08:00 after being woken at 06:30 following the first sleep period (Fig 1). The subject was asked to lie quietly on the bed undertaking no activity and not going to sleep. The RMR was calculated as the average metabolic rate for the period 07:20–07:50 when participants were least restless. As with the SMR, the values would be expected to vary with the weight of lean tissue and so the analysis included both raw values and values normalised to this.

### Metabolic rate during exercise (EMR-80,EMR-100, EMR-120)

The energy expenditure for each of the stepping exercises, the exercising metabolic rate (EMR) was calculated as the mean energy expenditure throughout the 30-minute period with the energy expenditure values for the 80,100 and 120 step min^-1^ exercises, denoted EMR-80, EMR-100 and EMR-120 respectively. The energy expenditure for the stepping exercise will depend on the total weight of the participant and therefore both raw values and values normalised to the total body weight were analysed.

### Diet induced thermogenesis (DIT)

DIT is the energy expended in the absorptive phase following feeding. This was calculated using the intercept method [27, 28]. The gradient, *a*, and intercept, *c*, are first determined for the line of best fit between the natural logarithm of the energy expenditure, *EE*_1_ and the movement sensor *M*_*s*_ for the 24 hour measurement period such that:

$$Ln({EE}_{1})=aM_{s}+c$$

where the natural logarithm is used to linearise the relationship between the energy expenditure and the output from the movement sensor. The DIT is then calculated using the following equation:

$$DIT=\bar{SMR}-e^{c}$$

where $\bar{SMR}$ is the mean energy expenditure from the two periods of sleep during the study.

### Statistical analysis

As the number of participants was small it was not possible to determine the normality of the data. Therefore, summarised values for the group of participants are presented as median and interquartile range (IQR). In this project we have studied the change in metabolism from measurements taken pre- and post the expedition. Where appropriate, the median and IQR of the post-expedition minus pre-expedition values are also reported. Statistical analysis of pre- to post-expedition measurements was performed on 5 participants and group comparisons have used the non-parametric sign test with statistical significance taken as *P* < 0.05. It should be noted that the test statistic for the Sign test is an integer and P < 0.05 is only achieved when the test statistic is zero which only occurs when a change in a measure for all the participants is in the same direction. This condition gives an exact probability of P = 0.03 which is the value used throughout the text. We have also analysed the changes for individual participants and how these varied across the group.

## Results

Five of the six reservists, including their leader, completed the traverse of Antarctica from the Hercules Inlet to the Ross Sea Ice. The sixth reservist left the expedition at the South Pole due to extreme fatigue and thus their data was excluded from the analysis. Table 1 shows the body composition for the other five participants from the pre- and post-expedition studies.

**Table 1:** The pre- and post-expedition values for body composition.

	Pre-expedition	Post-expedition
body weight /kg	85.8 (1.3)	80.2 (4.3)*
lean tissue weight /kg	74.1 (2.3)	73.1 (1.8)
fat weight /kg	12.2 (10.6)	5.7 (6.7)*

Statistical significance at *P<*0.05 is denoted *.

There was a median (IQR) reduction in the fat tissue from 12.2 (10.6)kg to 5.7 (6.7)kg, a decrease of 53%. This change was statistically significant at *P = 0*.*03*. There was a minimal, non-statically significant change in the average lean tissue weight, but the drop in fat weight gave an overall drop in body weight from 85.8(1.3) kg to 80.2 (4.3) kg, a decrease of 7%, that was statistically significant (*P* = 0.03).

**Fig 3:**
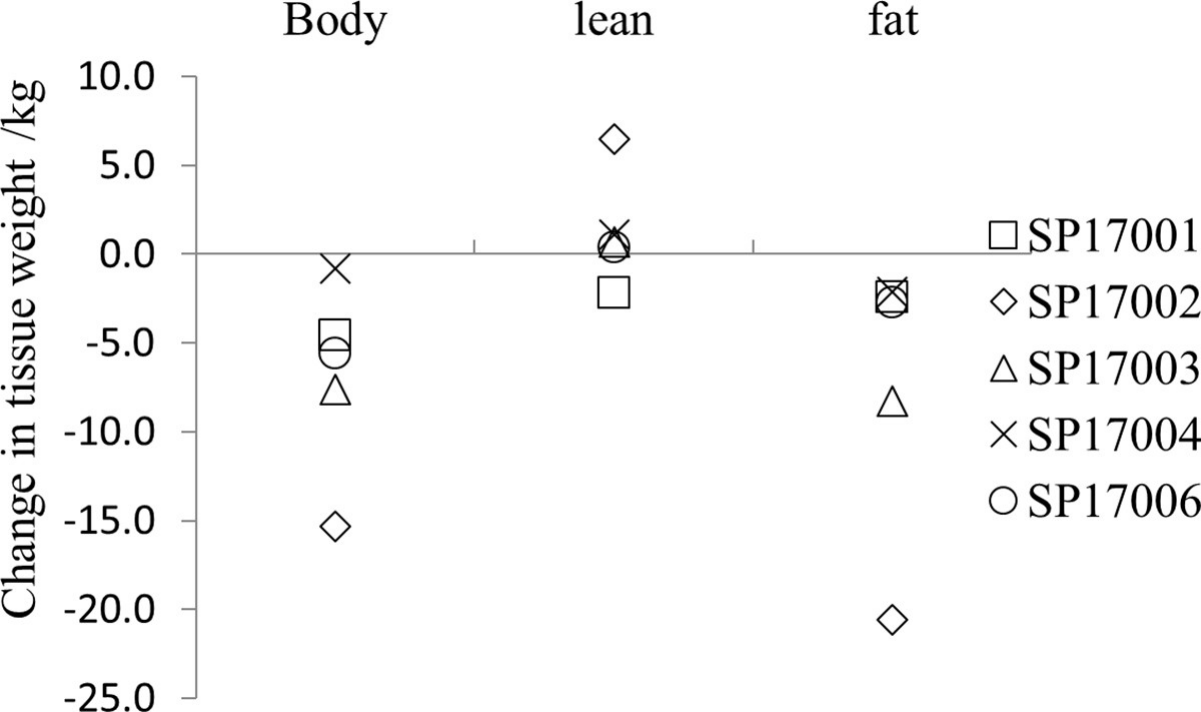
The change in body composition (post-expedition–pre-expedition).

From Fig 3 it can be seen that with one exception (SP17002) the changes in lean weight were small, but it is interesting to note that all but one participant (SP17001) showed an increase in the weight of lean tissue, including SP17002.

### Total energy expenditure (TEE)

During the measurement period, the average total energy expenditure (TEE) for the group was 3123 (196) kcal day^-1^ for the pre-expedition study and 2871 (128) kcal day^-1^ for the post expedition study, an 8% decrease. The change in TEE was not statistically significant as only four of the five participants had a decrease. The substrate utilisation across the measurement period for the group is given in Table 2.

**Table 2:** Average [median (IQR)] substrate utilisation pre- and post-expedition for the during the 24hour measurement period.

	Pre-expedition	Post-expedition
Protein /g day^-1^	99 (12)	102 (4)
Carbohydrate /g day^-1^	330 (46)	333 (92)
lipid /g day^-1^	116 (31)	110 (46)

**Fig 4:**
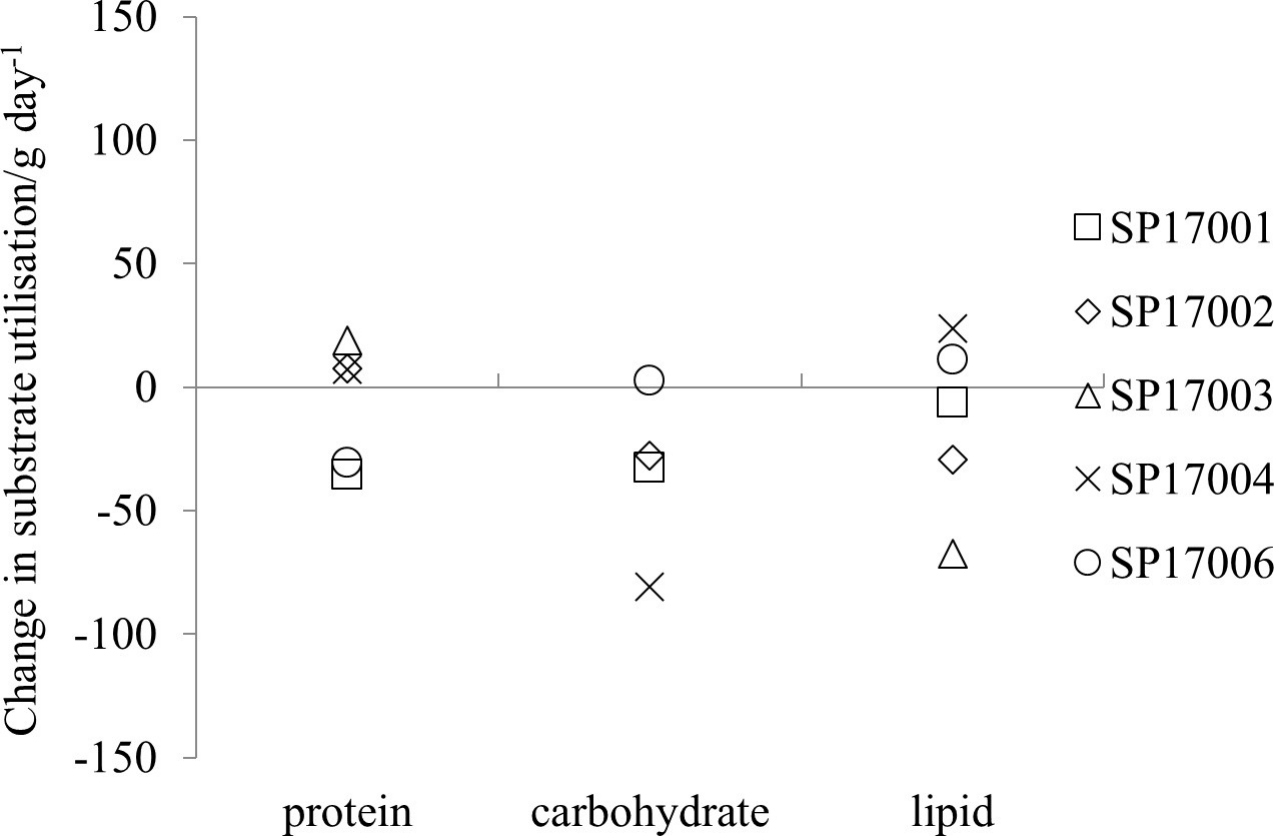
The change in substrate utilisation in g/day for the 24-hour measurement periods.

The difference in substrate utilisation between the post- and pre-expedition measurements for the individual participants (Fig 4) shows that there was either no-change or a reduction in carbohydrate utilisation for the post-expedition study but the direction of changes of protein and lipid utilisation is not consistent across participants. The average reduction in carbohydrate utilisation between the pre- and post-expedition measurements was -28 (35) g day^-1^. None of the changes in substrate utilisation determined for the whole measurement period were statistically significant.

### Sleeping metabolic rate (SMR)

**Fig 5:**
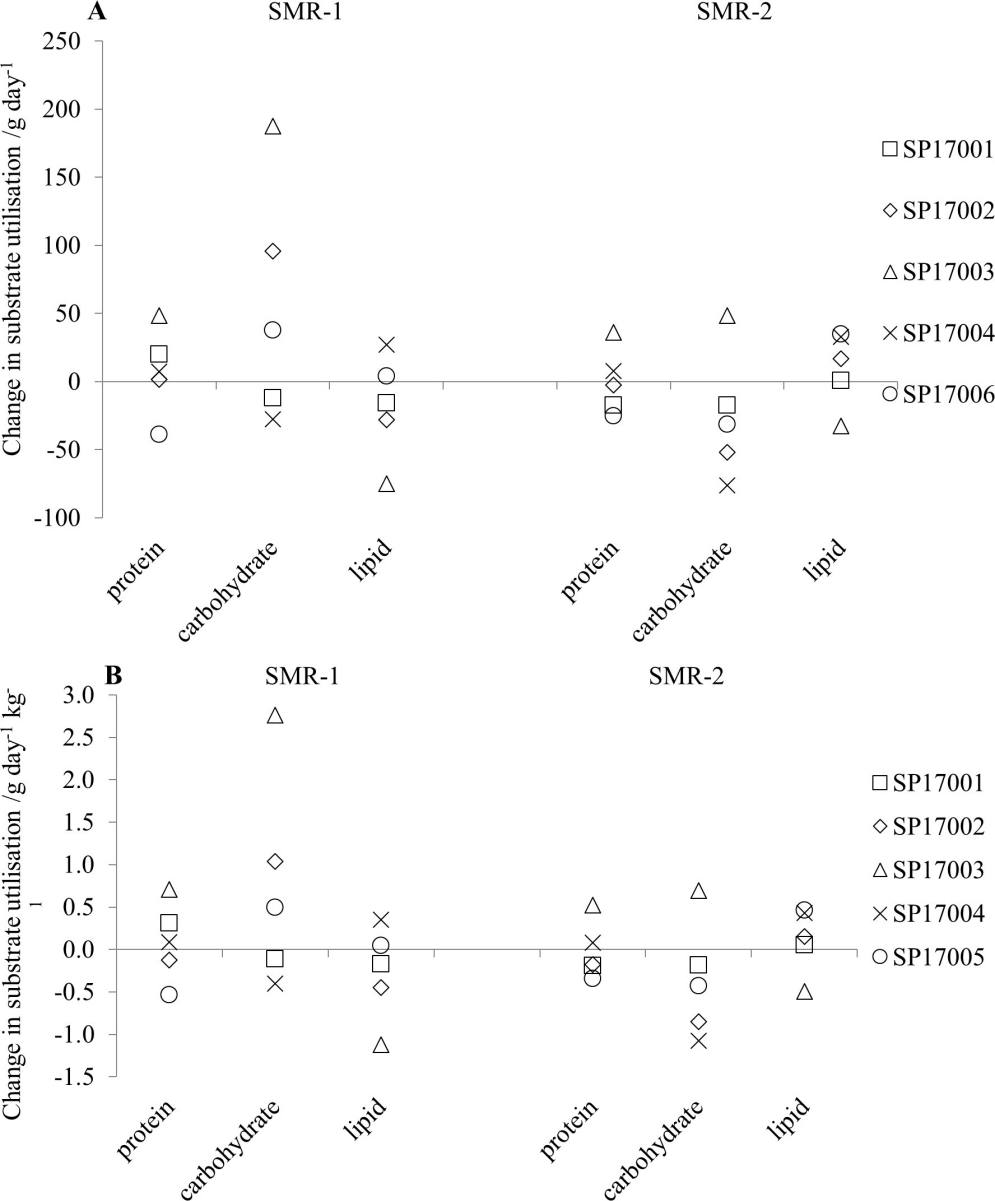
The change in substrate utilisation (post-expedition–pre-expedition) during the two sleep periods (SMR-1 and SMR-2). (A) raw values; (B) the values normalised to lean tissue weight.

The metabolic rate values for the first and second periods of sleep are 1.39 (0.13) kcal min^-1^ and 1.34 (0.05) kcal min^-1^, respectively, for the pre-expedition measurements and 1.40 (0.03) kcal min^-1^ and 1.34 (0.05) kcal min^-1^, respectively for the post-expedition measurements. None of these differences are statistically significant. The average substrate utilisation during sleep for the group is given in Table 3 and the change from the pre-expedition values in Fig 5A for individual participants.

**Table 3:** Average [median (IQR)] substrate utilisation pre- and post-expedition for the energy expenditure during the two periods of sleep, SMR-1 and SMR-; raw and normalised to lean tissue weight.

	SMR-1		SMR-2	
	Pre-exp	Post-exp	Pre-exp	Post-exp
Protein /g day^-1^	91 (39)	108 (37)	121 (8)	132 (29)
Carbohydrate /g day^-1^	130 (8)	125 (117)	141 (9)	123 (45)
lipid /g day^-1^	94 (17)	102 (49)	87 (21)	100 (30)
Protein /g day^-1^ kg^-1^ lean tissue	1.4 (0.5)	1.5 (0.3)	1.6 (0.2)	1.6 (0.4)
Carbohydrate /g day^-1^ kg^-1^ lean tissue	1.8 (0.1)	1.7 (1.3)	2.1 (0.2)	1.7 (0.4)
lipid /g day^-1^ kg^-1^ lean tissue	1.4 (0.2)	1.4 (0.7)	1.2 (0.4)	1.4 (0.5)

From the table it can be seen that there is a much higher variability in the carbohydrate utilisation across the participants in the first sleep period when compared to the second sleep period and this is seen in both the pre- and post-expedition measurements. In Fig 5A it can be seen that there is also a much greater range of values for the difference in carbohydrate utilisation between the pre- and post-expedition measurements in the first sleep period when compared with the second. The average difference between the pre- and post-expedition carbohydrate utilisation was 37 (107) g day^-1^ for SMR-1 and -31 (24) g day^-1^ for SMR-2. Thus, when compared with the pre-expedition values, the post-expedition values show an increase in carbohydrate utilisation during the first sleep period and a reduced carbohydrate utilisation during the second sleep period. Neither of these changes is statistically significant. These changes are not seen in either the protein or the lipid utilisation. However, this pattern of change in substrate utilisation is still seen when the metabolic rate values are normalised to the lean tissue weight (Fig 5B). When normalised in this way, the metabolic rate values for the first and second periods of sleep pre- and post the expedition are 0.02 (0.001) kcal min^-1^ and 0.02(0.0002) kcal min^-1^ respectively for the pre-expedition measurements and 0.02 (0.03)kcal min^-1^ and 0.02 (0.001) kcal min^-1^, respectively, for the post-expedition measurements. The average values for substrate utilisation pre- and post- the expedition are given in Table 3. None of the differences between the pre- and post-expedition values for normalised substrate utilisation were statistically significant.

### Resting metabolic rate (RMR)

**Fig 6:**
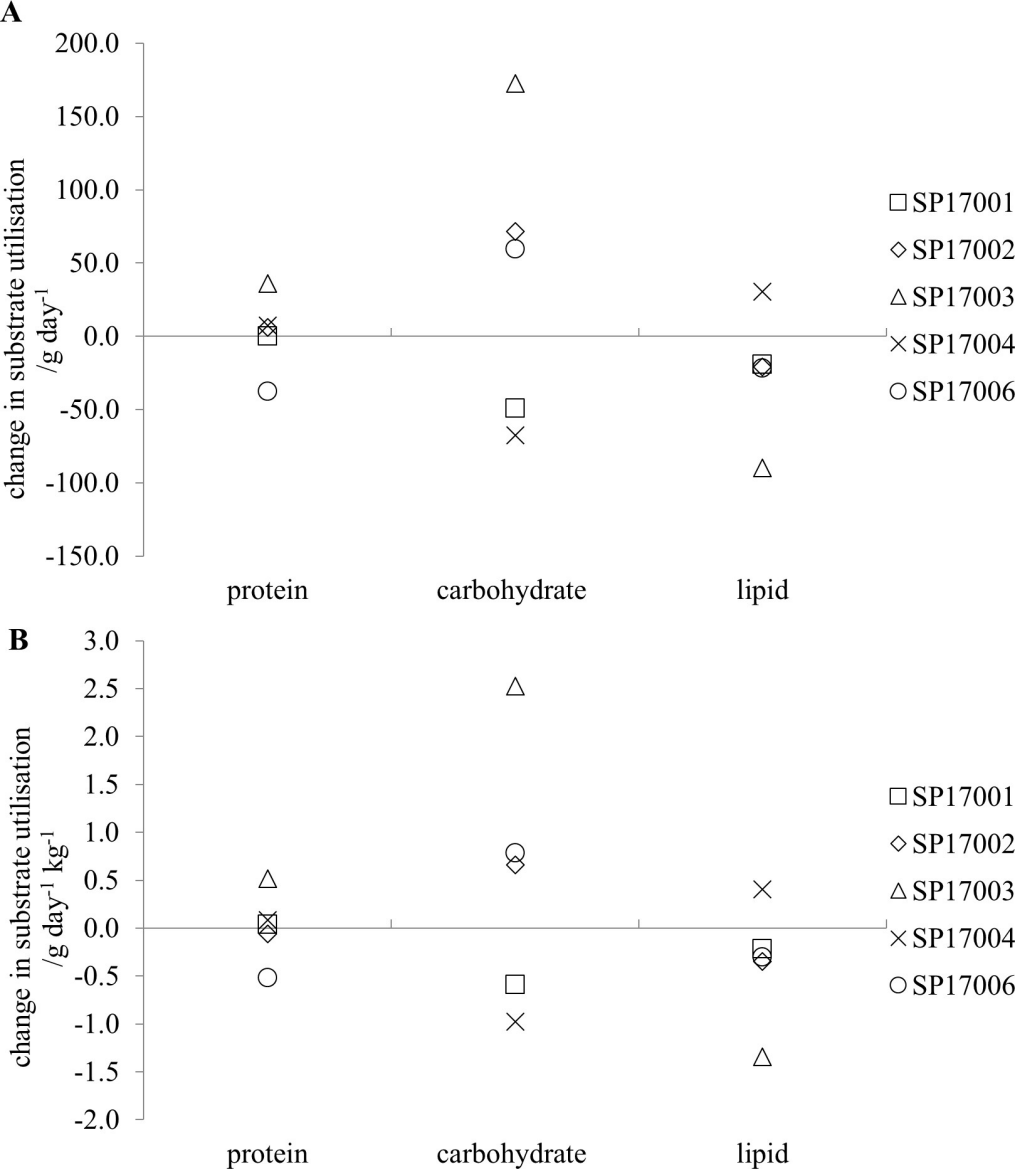
The change in substrate utilisation during measurement of the resting metabolic rate (RMR) (post-expedition–pre-expedition). (A) raw values; (B) the values normalised to lean tissue weight.

The mean values for the pre- and post-expedition measurements of the Resting Metabolic Rate (RMR) were 1.42 (0.10) kcal min^-1^ and 1.40 (0.06) kcal min^-1^, respectively with the average values for substrate utilisation given in Table 4. In terms of these average values there was minimal change in the average utilisation of any of the substrates, although there was a 10% reduction in the lipid utilisation between the pre- and post-expedition measurements, but the direction of the change was not consistent across participants and therefore not statistically significant (Fig 6A). As with the SMR results, this figure shows a large range in carbohydrate utilisation across different members of the group with a much smaller spread of values for protein utilisation. With the exception of SP17003, there was only a small change in the lipid utilisation between the pre- and post-expedition measurements. SP17003 had a much larger reduction in lipid utilisation than the other participants, but also the largest increase in carbohydrate utilisation. The average difference between the pre- and post-expedition carbohydrate utilisation was 60 (120) g day^-1^ for the RMR. When normalised to the weight of lean tissue, the mean values for RMR pre- and post-expedition are: 0.02 (0.0006) kcal min^-1^ and 0.02 (0.0002) kcal min^-1^, respectively.

**Table 4:** Average [median (IQR)] substrate utilisation pre- and post-expedition for the resting metabolic rate (RMR); raw and normalised to lean tissue weight.

	RMR	
	Pre-exp	Post-exp
Protein /g day^-1^	105 (30)	105 (30)
Carbohydrate /g day^-1^	209 (55)	209 (83)
lipid /g day^-1^	94 (19)	84 (31)
Protein /g day^-1^ kg^-1^ lean tissue	1.4 (0.3)	1.4 (0.3)
Carbohydrate /g day^-1^ kg^-1^ lean tissue	2.8 (0.5)	2.8 (0.8)
Lipid /g day^-1^ kg^-1^ lean tissue	1.4 (0.3)	1.2 (0.5)

The changes in average substrate utilisation were maintained when the values were normalised to lean tissue weight (Table 4) with a large range of values for carbohydrate utilisation and a small range of values for protein utilisation. With the exception of SP17003 (Fig 6B), there is also a small range of values for the change in lipid utilisation.

### Metabolic rate during exercise (EMR-80, EMR-100 and EMR-120)

The average metabolic rate across the group for the three intensities of exercise pre- and post-expedition are given in Table 5. From this table it can be seen that there is the expected increase in energy expenditure as the step rate increased. The differences between the pre- and post-expedition values were small but all participants showed a reduction in energy expenditure at 80 steps min^-1^ that gave statistical significance between the pre- and post-expedition values. When the values for the 100 step min^-1^ and 120 step min^-1^ exercises were normalised to total body weight (Table 5) the direction of the change between the pre- and post-expedition values remained the same and the difference small. However, for the 80 step min^-1^ intensity, the direction of the change between the pre- and post-expedition values reversed, was no longer consistent across all participants and therefore was no longer statistically significant.

**Table 5:** Average [median (IQR)] energy expenditure during exercising (EMR) pre- and post-expedition for the three exercise intensities.

	Exercise metabolic rate	
	Pre-exp	Post-exp
80 steps min^-1^ (EMR-80) /kcal min^-1^	4.9 (0.1)	4.5 (0.1)*
100 steps min^-1^ (EMR-100) /kcal min^-1^	3.6 (2.2)	3.6 (1.6)
120 steps min^-1^ (EMR-120) /kcal min^-1^	6.5 (0.9)	6.4 (0.2)
80 steps min^-1^ (EMR-80) /kcal min^-1^ kg^-1^	0.1 (0.002)	0.1 (0.003)
100 steps min^-1^ (EMR-100) /kcal min^-1^ kg^-1^	0.04 (0.02)	0.05 (0.018)
120 steps min^-1^ (EMR-120) /kcal min^-1^ kg^-1^	0.1 (0.009)	0.1 (0.004)

Statistical significance at *P<*0.05 is denoted *.

**Fig 7:**
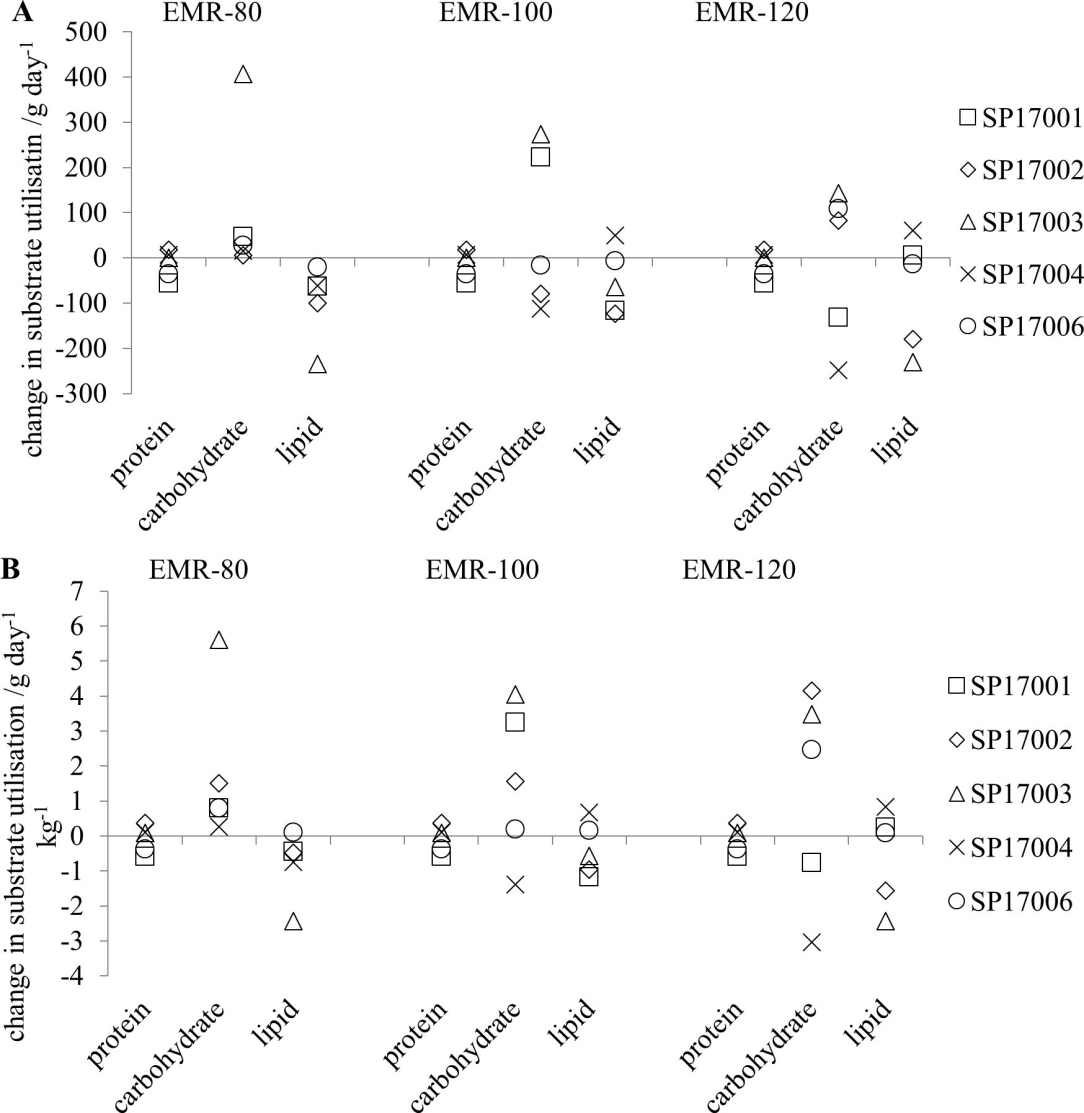
The change in substrate utilisation during the three periods of exercise (post-expedition–pre-expedition) where EMR-80, EMR-100 and EMR-120 are the measurements at 80 steps min^-1^, 100 steps min^-1^ and 120 steps min^-1^, respectively. (A) raw values; (B) the values normalised to body weight.

The average substrate utilisation rates for the three intensities (Table 6) show a statistically significant increase in carbohydrate utilisation and a statistically significant decrease in protein utilisation for the 80 step min^-1^ exercise intensity between the pre- and post-expedition values. It should be noted that the protein values are the same for all exercise periods, as a 12-hour sample period covered all three exercise sessions. The average change in carbohydrate utilisation values for the three exercise intensities were: 28 (31) g day^-1^, -17 (302) g day^-1^ and 83 (241) g day^-1^ for EMR-80, EMR-100 and EMR-120, respectively. Whilst there was an average increase in the carbohydrate utilisation and reduction in the protein utilisation for the other exercise intensities, the direction of the change was not consistent across the different participants (Fig 7A) and so was not statistically significant. When the values were normalised to body weight (Table 6), the increase in carbohydrate utilisation between the pre- and post-expedition values was still statistically significant but the reduction in lipid utilisation was not. For both raw and normalised differences between the pre- and post-expedition substrate utilisation (Fig 7A and 7B, respectively) the range of values for the change in lipid utilisation was much smaller than for the other substrates. It should be noted that one of the participants, SP17003, had a much higher carbohydrate utilisation for the 80 step min^-1^ intensity than the other participants which is not seen at the higher exercise intensities (Fig 7A and 7B).

**Table 6:** Average [median (IQR)] substrate utilisation pre- and post-expedition for the three exercise intensities where EMR-80, EMR-100 and EMR-120 are the measurements whilst exercising at 80 steps min^-1^, 100 steps min^-1^ and 120 steps min^-1^, respectively; unaltered and normalised to total body weight.

	EMR-80		EMR-100		EMR-120	
	Pre-expedition	Post-expedition	Pre-expedition	Post-expedition	Pre-expedition	Post-expedition
Protein /g day^-1^	79 (14)	76 (27)	79 (14)	76 (27)	79 (14)	76 (27)
Carbohydrate /g day^-1^	540 (180)	569 (195)*	635 (355)	746 (548)	1451 (313)	1458 (180)
lipid /g day^-1^	450 (61)	388 (127)*	286 (56)	212 (67)	318 (45)	259 (71)
Protein /g day^-1^ kg^-1^	0.9 (0.06)	1.0 (0.28)	0.9 (0.06)	1.0 (0.28)	0.9 (0.06)	1.0 (0.28)
Carbohydrate /g day^-1^ kg^-1^	6.3 (2.88)	7.4 (2.18)*	8.2 (3.99)	9.5 (6.13)	16.9 (1.27)	18.5 (2.21)
lipid /g day^-1^ kg^-1^	5.7 (0.75)	5.1 (1.69)	2.9 (0.65)	2.8 (0.69)	3.2 (0.57)	3.4 (0.72)

Statistical significance at *P<*0.05 is denoted *.

### Diet induced thermogenesis (DIT)

The mean values for DIT pre- and post-expedition were 384(141) and 226(100) kcal day^-1^ respectively for a pre-expedition nutritional intake of 2956(178) kcal day^-1^ and a post-expedition nutritional intake of 2856(103) kcal day^-1^. The change in DIT was substantial between the pre- and post-expedition measurements. All participants gave a lower post-expedition value when compared with the pre-expedition value and therefore the change was statistically significant. The DIT as a percentage of the nutritional intake for the pre- and post-expedition studies was 12.2% and 7.7%.

## Discussion

The energy expenditure and substrate utilisation were measured using a whole body calorimeter on five participants pre- and post- an Antarctic expedition. The carbohydrate and lipid utilisation were calculated from the difference in the concentrations of O_2_ and CO_2_ entering and leaving the chamber, and the protein utilisation determined from analysis of the urine samples. These analyses give values in units of g day^-1^. Using values for the energy density for each substrate of 4.2kcal g^-1^ for carbohydrate, 9.4kcal g^-1^ for lipid and 4.7kcal g^-1^ for protein [29], the sum of the energy from the three substrates during the measurement period was within 2% of the calculated total energy expenditure (TEE) value.

Perhaps the most striking and surprising finding in this study was the modest loss of body weight, approximately 7% between the pre-and post-expedition measurements. Whilst the reduction in body weight was statistically significant, that significance comes from all the participants losing weight rather than the magnitude of the weight loss. All the weight loss comes from fat loss, a change that was also statistically significant, and there was an increase in lean weight for all but one of the participants. These findings are consistent with other studies on those undertaking expeditions [5, 9, 10]. However, these changes in body weight and composition contrasts with that reported by Stroud *et al*. (3) for two males who undertook an unsupported 48 day 810km Arctic expedition who lost 7.1kg and 5.9kg of fat tissue and 5.7 and 7.6kg of lean tissue. The same two males who undertook a 95day, 2300km Antarctic expedition lost 10.2kg and 12.3kg of body weight [4]. Factors affecting the energy expenditure during an expedition include the air temperatures, the terrain and the sledge weight. Stroud *et al*. (4), give the weight of the sledges pulled in Antarctica as 222kg. However, in books about these expeditions[12, 30] a description is given implying a starting sledge weight of around 222kg but ‘dumping’ supplies and equipment to lighten the load of the sledges early in the expedition is mentioned. Unfortunately, neither author gives any indication of the likely weight removed. The planned weight of sledges pulled by the Spear-17 participants was 120kg. However, the fatigue of the sixth member of the Spear-17 expedition led to weight from his sledge being re-distributed amongst other participants, although not equally. It must also be recognised that the Spear17 had a single re-supply at the pole, whilst other expeditions, did not; therefore, reducing the sledge weight and therefore the physical activity demand on the expedition study in this paper. A small loss of body fat has been reported in men over-wintering in Antarctica at 2,750m who were only undertaking ‘heavy work’ for less than an hour a day and whose diet was within recommended daily levels for fat, protein and carbohydrate [11]. It was suggested that this decrease was due to changes in substrate utilisation in response to chronic hypoxia.

The rations for the Spear-17 expedition were 6,500 kcal/participant/day. Assuming the median fat loss of 6.5kg (Table 1) was achieved linearly over the 67days of the expedition, a daily fat loss of 97g is obtained. Using a calorific value for fat of 9.4kcal g^-1^ [29] and ignoring the lean tissue gain which is small, suggests a daily Energy Availability deficit of 912kcal giving a daily energy expenditure of 6500+912 = 7412kcal. This is close to the energy expenditure values of 6740kcal day^-1^ and 7739 kcal day^-1^ measured using DLW in two participants during an Arctic expedition [3] and 6381 kcal day^-1^ and 8700 kcal day^-1^ measured on the same two participants by the same technique during an Antarctic expedition [4]. However these values are substantially higher than the 6100 kcal day^-1^ estimated using activity patterns [15]. Unfortunately, due to constraints placed on the research by the expedition organisers and their support partners, DLW was not measured during the Spear-17 expedition. Stroud (2) suggested there was a 60% increase in basal metabolic rate during polar expeditions. The values of metabolic rate obtained in this study during resting (RMR) and sleeping (SMR-1, SMR-2), which are measures of the body’s basal metabolic rate, were approximately 1.4kcal min^-1^ (2016kcal day^-1^) and no difference was found between the pre- and post-expedition measurements. 60% of this is 1210kcal day^-1^ which would explain the difference between the 6100 kcal day^-1^ activity estimate and the calculated energy expenditure during the Spear-17 expedition of 7412 kcal day^-1^. Stroud (2) attributed the 60% increase in basal metabolic rate (BMR) to thyroid activity based on observations of elevated heart rate, restlessness and heat intolerance. However, in evaluating the difference, the prolonged exposure to a cold environment, particularly during sleeping, cannot be ignored. The importance of this is supported by a weight loss in the male subjects over-wintering in Antarctica, who only undertook low levels of physical activity for the majority of time and whose nutrition was appropriate to the level of activity [11]. Using model based techniques to determine the energy required for thermoregulation, a 843kCal day^-1^ increase was found for those undertaking vigorous work in a cold climate (-7.5°C) when compared with a temperate climate (12°C) [31]. These figures support the proposed explanation that the increase in BMR is due to thermoregulation as temperatures down to -60°C occur during the Antarctic summer. This is also consistent with there being no change in the values measured pre- and post-expedition as the controlled environment of the chambers would create a minimal energy requirement for thermoregulation. Essentially, the Energy Availability for participants in the Spear-17 expedition was much higher than in previously reported expeditions [3, 4] with similar energy expenditure.

Whilst there was no difference between the energy expenditure measured pre- and post-expedition during resting and sleeping, a number of participants showed an increase in the carbohydrate utilisation, particularly during the first sleep period (SMR-1) and measurement of the resting metabolic rate (RMR)–changes that were not statistically significant because not all participants showed an increase in carbohydrate utilisation.

The difference between pre- and post-expedition measurement of energy expenditure during exercise was statistically significant for lowest step rate (80 steps min^-1^) but not for the higher step rates (100 step min^-1^ and 120 step min^-1^). The 80 step min^-1^ exercise was performed whilst the participants were fasted and therefore is the most likely to reflect an underlying physiological change due to the expedition. Analysis of the substrate utilisation showed that the difference at 80 steps min^-1^ was due to an increase in carbohydrate utilisation in the post-expedition measurements, which was accompanied by a reduction in lipid utilisation. However, there was no systematic change in the utilisation of any of the substrates as the exercise intensity increased. Although the difference in carbohydrate utilisation between the pre- and post-exercise measurements at 80 steps min^-1^ is maintained when normalised to body weight, the large range of pre- to post-expedition differences in carbohydrate utilisation across all exercise intensities suggests that a change in carbohydrate metabolism did occur in some participants, but not others.

The total energy expenditure (TEE) measures the metabolic energy required to deliver the protocol during the measurement period. Whilst this contains specified activities including eating, sleeping and the step exercises the remaining time was taken up with unspecified very low-level activity. These activities were matched between the pre- and post-expedition measurements through detailed laboratory records of the subject’s activity. However, there was an 8% reduction in the measured energy expenditure between the pre- and post-expedition measurements. All participants, with the exception of SP17003, showed a reduction in energy expenditure. Without SP17003 the change in TEE would have been statistically significant. The analysis of the substrate utilisation shows that this difference is largely due to a reduction in carbohydrate utilisation.

This finding of reduced carbohydrate utilisation during the 24 hour measurement period appears to be inconsistent with the increased in carbohydrate utilisation found during the first sleep period, resting and during the lowest intensity exercise. However, the increase in carbohydrate utilisation was only seen during the first sleep period that is not part of the 24-hour measurement period and the lowest intensity exercise lasted only 30 minutes (2%) of the 24-hour measurement period. A decrease in carbohydrate utilisation between the pre- and post-expedition measurements was found for the second period of sleep. The majority of the time the participant was awake during the 24-hour measurement period was spent in very low-level physical activity. Carbohydrate utilisation during the measurement period is a time-averaged value and therefore carbohydrate utilisation during sleep and the low-level activity is smaller in the post-expedition measurements than in the pre-expedition measurements. It should be noted that during measurement of the RMR there was a large increase in carbohydrate utilisation for three of the five participants when the post-expedition measurements were compared with the pre-expedition measurements. It should further be noted that like the 80 step min^-1^ exercise, the RMR measurement was made whilst the participants were fasted. Increased glucose utilisation has been reported during the exposure of male participants and animals to moderate altitudes (4300m) [32, 33], but this is not a universal finding as a recent study, albeit at a lower altitude (3375m), did not find an increase in glucose utilisation [34]. We have identified a reduced utilisation of carbohydrate in the post-expedition measurements when compared with the pre-expedition measurements on participants in the Spear-17 expedition. The maximum altitude attained during the Spear-17 expedition was 3350m but without data on energy expenditure and substrate utilisation during the expedition itself we cannot investigate the origin of these changes further, specifically whether there was a change with a change in altitude. Increased carbohydrate utilisation has been found in resting subjects exposed to cold conditions at sea level for a short period; a change that was accompanied by an increase in lipid utilisation [35]. However, the cold exposure only lasted 2 hours and the temperature reduction was only down to 10°C so the environmental conditions were not comparable with those experienced by the Spear-17 participants.

The intercept method of determining the diet induced thermogenesis (DIT) assumed a 2-component model of energy expenditure where the total energy expenditure is the sum of an activity independent component and an activity dependent component where there the relationship between energy expenditure and activity is linear, or can be transformed to be linear. The spread of data around the line-of-best-fit used to describe the energy dependent component gives an uncertainty in the intercept that means values of DIT determined by this method must be treated with caution. The DIT is between 10% and 15% of the calorific value of the dietary intake, depending on the composition of the diet [36] and the composition of the diet was the same for both the pre- and post-expedition measurements. The pre- and post-expedition values obtained in this study represent 12.2% and 7.7% of the dietary intake. However, with the exception of SP17003, the change in the calorific value of the diet during the measurement period was less than 3% between the measurements and not all changes were in the same direction. All the participants showed a decrease in DIT when the post-expedition measurement was compared with the pre-expedition measurement and it was this that gave statistical significant. A reduction in DIT between the pre- and post-expedition measurements is consistent with the drop in energy expenditure found for the TEE.

Our aim was to study the impact of the expedition on energy expenditure and substrate utilisation. This includes acclimatization to extremely low ambient temperatures [37], altitude as well as the high levels of physical work undertaken for up to 10 hours each day [38]. The majority of the pre-expedition training was done in the UK with only two short periods of training in low altitude cold environments (Norway and Iceland). Work on recovery from extended exposure to polar climates, whilst undertaking high workloads, suggests that body composition returns to pre-expedition levels within a couple of weeks [9] but hormones can take 5 weeks to return to pre-expedition levels [39]. Both short duration pre-expedition training sessions outside the UK took place more than 5 weeks before the pre-expedition whole-body calorimetry study and so any acclimatization should have been reversed. Therefore, acclimatization to the cold and altitude occurred during the expedition itself. The time course for acclimatization is component dependent with the presence and activation of brown adipose tissue on exposure to cold temperatures being found after 10 days whilst an increase in haemoglobin is found a few days after exposure to altitude [37, 38]. Since these changes are over much shorter periods than the duration of the expedition, it was assumed that acclimatisation changes were complete well before the end of the expedition.

There was also a delay between the participants completing the expedition and the post-expedition whole-body calorimeter study. Although this was kept to a minimum, unrestricted availability of palatable food and only voluntary exercise led to the body composition changes we report which are consistent with the principle of reversibility [40]. The participants with also have experienced de-acclimatization on returning to lower altitude. This is not a well-studied phenomenon and although it clearly occurs, the timescale over which this happens is unknown and is likely to be variable between individuals [41]. It should also be noted that the altitude achieved on the Antarctic plateau is relatively modest at 3800m and so both acclimatisation and de-acclimatisation processes are likely to be somewhat attenuated compared to expeditions at high-altitudes. Similarly research into the short term effects (< 4weeks) of detraining is also limited; in the case the Spear17 participants detraining equates to the cessation of sledge-hauling. Our finding of no change in resting metabolic rate agrees with the findings of Laforgia *et al*. [42]. However, our finding of increased carbohydrate utilisation during resting and low-level exercise in the post-expedition measurement is inconsistent with the findings of Mujika and Padilla [43] who report an increased reliance on carbohydrate metabolism at maximal and sub-maximal exercise. Without a detailed record of the participant’s consumption and activity, caution is necessary in including detraining in the interpretation of the energy expenditure and substrate utilisation measures. In addition, detraining effects are known to vary according to numerous factors including starting condition and age [44]; therefore, an intra-subject variability in detraining effect is potentially a further source of variance in the measured data. In addition to activity related dynamics, the dietary and environmental factors must also be considered. Given participants were in a different environment, it can be assumed that the diet would not have been indicative of their habitual diet in terms of macronutrient or consumption patterns; this would be replicated in the physical activity, being greater in sedentary behaviour with no high-intensity training. Whilst Punta Arenas, where the participants spent 10 days before returning to the UK, is slightly cooler than the UK, the geographic home for all members of the Spear-17 expedition, it is still a moderate climate at sea-level, enforcing little additional energy load to maintain thermal equilibrium.

The findings reported in this paper not only have implications for those planning, supervising, and undertaking polar travel but also for expeditionary travel in other environments, for endurance athletes, for military combat training and in the nutritional provision to the survivors of disasters in extreme environments. The role of adequate nutrition in improving healthcare outcomes is also recognised [45] and the results reported in this paper on substrate utilisation, particularly carbohydrate utilisation, support investigating whether nutritional composition affects healthcare outcomes.

This study has demonstrated only small changes in energy utilisation as a result of an extended Antarctic expedition involving high workloads in very low temperatures. The small loss of body weight, in contrast to that reported in other studies, is evidence of adequate and appropriate nutrition throughout the expedition itself. Our results suggest that following the expedition there is a decrease in carbohydrate utilisation when fed but an increase in carbohydrate utilisation when fasted. However, it is impossible using only pre- and post-expedition data to determine the origin of this and, as a minimum, data on energy expenditure during the expedition, or from long duration experiments in an equivalent simulated environment, are required to understand fully the origin of these findings. Whilst there are differences between the pre- and post-expedition measurements it must be stressed these are small–the expedition had minimal impact on the metabolic energy requirements of the participants. Essentially the selection, training regime and the composition and quantity of the nutrition during the expedition allowed the team to successfully complete the expedition. There is anecdotal evidence from the expeditionary team that the member of the expedition who left it at the South Pole due to extreme fatigue had, ceased following the training regime and gained a large amount of weight before departure. Thus suggesting that when appropriately selected and prepared, arduous expeditions can be undertaken with minimal long-term impact on the energy requirements of the participants.

## Abbreviations

EA: Energy availability
DLW: Doubly Labelled Water
IQR: Interquantile Range
ADP: Air Displacement Plethysmography
TEE: Total Energy Expenditure
SMR: Sleeping Metabolic Rate
RMR: Resting Metabolic Rate
EMR: Exercising Metabolic Rate
DIT: Diet Inducted Thermogenesis
